# Synthesis of ammonia using sodium melt

**DOI:** 10.1038/s41598-017-12036-9

**Published:** 2017-09-14

**Authors:** Fumio Kawamura, Takashi Taniguchi

**Affiliations:** Research Center for Functional Materials, High Pressure Group, National Institute for Materials Science (NIMS), 1-1 Namiki, Tsukuba, Ibaraki, 305-0044 Japan

## Abstract

Research into inexpensive ammonia synthesis has increased recently because ammonia can be used as a hydrogen carrier or as a next generation fuel which does not emit CO_2_. Furthermore, improving the efficiency of ammonia synthesis is necessary, because current synthesis methods emit significant amounts of CO_2_. To achieve these goals, catalysts that can effectively reduce the synthesis temperature and pressure, relative to those required in the Haber-Bosch process, are required. Although several catalysts and novel ammonia synthesis methods have been developed previously, expensive materials or low conversion efficiency have prevented the displacement of the Haber-Bosch process. Herein, we present novel ammonia synthesis route using a Na-melt as a catalyst. Using this route, ammonia can be synthesized using a simple process in which H_2_-N_2_ mixed gas passes through the Na-melt at 500–590 °C under atmospheric pressure. Nitrogen molecules dissociated by reaction with sodium then react with hydrogen, resulting in the formation of ammonia. Because of the high catalytic efficiency and low-cost of this molten-Na catalyst, it provides new opportunities for the inexpensive synthesis of ammonia and the utilization of ammonia as an energy carrier and next generation fuel.

## Introduction

Ammonia is an important raw material for synthesizing chemical fertilizer, and has supported worldwide food production since the development of the Haber-Bosch process^1^. In recent years, ammonia has also attracted attention as an energy carrier and a next generation fuel^2–4^. For instance, Hydrogen transport can be facilitated by conversion into ammonia. Furthermore, ammonia is a promising environmental friendly fuel because the combustion of ammonia does not produce CO_2_.

Typically, ammonia is produced using the Haber-Bosch process, which relies on an Fe-based catalyst. However, this process requires a high reaction temperature (400–600 °C) to dissociate the triple bond of nitrogen molecules (945 kJ/mol)^5^ and a high pressure (20–40 MPa) to suppress the decomposition of ammonia synthesized at high temperature. Maintaining high temperatures and pressures increases the production cost of ammonia and prevents its use as an energy carrier or fuel.

In 1972, a Ru-based catalyst was reported for ammonia synthesis under milder conditions than those required in the Haber-Bosch process^6^. Since then, Ru-based catalysts have been researched intensively^7^ This research has led to the development of an Ru-loaded electride catalyst^8, 9^ for the synthesis of ammonia. With this catalyst, the rate-limiting reaction is the formation of N-H_n_ species on the surface of the catalyst, rather than the decomposition of the nitrogen triple bond^10^.

Development of catalysts based on inexpensive elements has also been undertaken. Co_3_Mo_3_N and Co-Mo bimetal have recently been reported as effective catalysts, with activities comparable to those of Ru-based catalysts^11–13^.

The electrocatalytic synthesis of ammonia has also been researched extensively because these syntheses may be conducted under atmospheric pressure^14–19^. In electrocatalytic syntheses, hydrogen is ionized at an anode and the resulting protons travel through an electrolyte to react with nitrogen on a cathode to form ammonia. In addition, attempts have been made to use water at the hydrogen source for electrocatalytic ammonia synthesis^20–26^.

**Figure 1:**
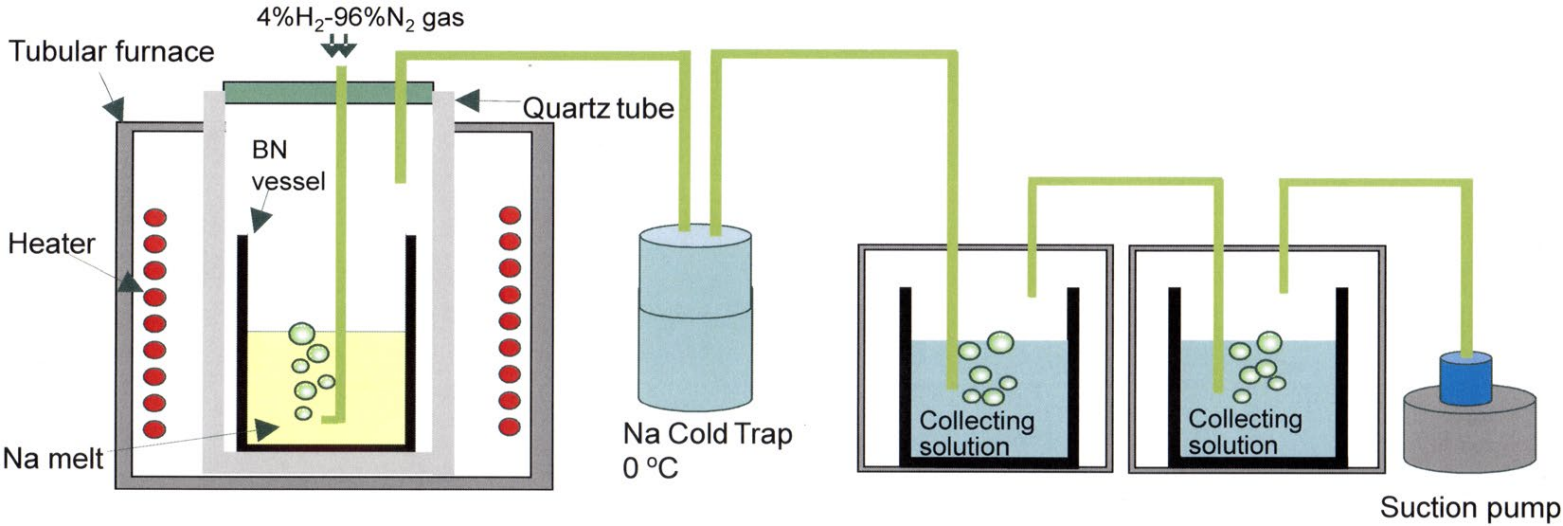
Schematic illustration of ammonia synthesis using a Na-melt. Na vapor is trapped in a cold-trap before reaching the collecting solution.

In the present study, we found that Na metal is capable of dissociating nitrogen molecules and developed an ammonia synthesis method based on bubbling H_2_-N_2_ mixed gas through a Na-melt at atmospheric pressure (Fig. 1).

## Results and Discussion

**Figure 2:**
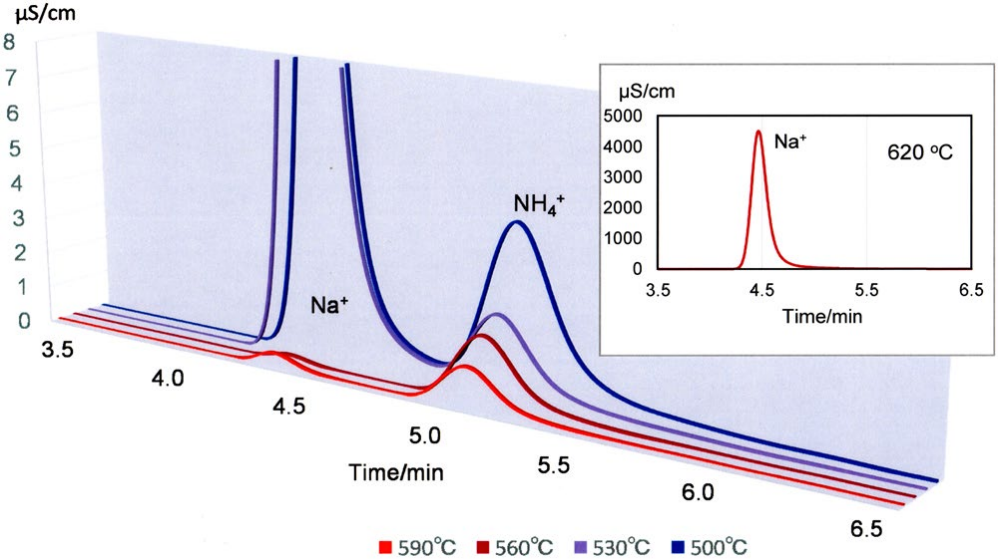
Ion chromatograms of collecting solutions. The maximum Na^+^ values obtained after reactions at 500 and 530 °C are 21 and 32 μS/cm, respectively. The inset shows the ion chromatogram of the collecting solution after the experiment at 620 °C, because the maximum value of Na^+^ is too large to indicate with the other chromatograms.

**Figure 3:**
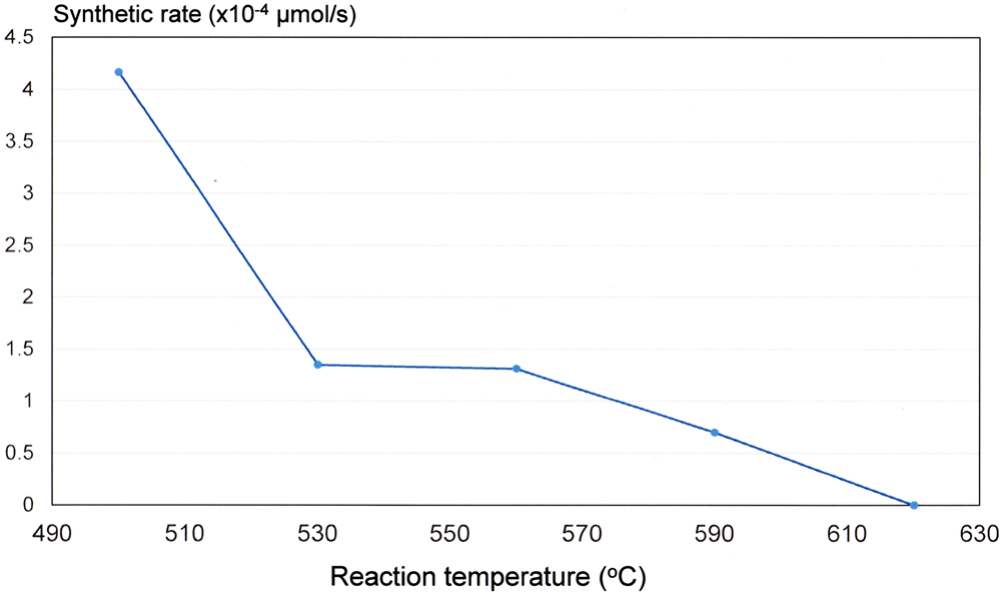
Synthetic rate of ammonia at reaction temperatures of 500–620 °C.

Figures 2 and 3 show ion chromatograms of the collecting solution and the reaction rate of ammonia, respectively, at various temperatures. These results were based on the ammonia collected after passing 4%H_2_-96%N_2_ gas through the Na-melt at a rate of 200 sccm for 20 min.

NH_4_^+^ and Na^+^ were clearly detected in the collecting solutions of experiments conducted at 500–590 °C. The maximum synthetic rate of ammonia (4.17 × 10^−4^ μmol/s) was observed at 500 °C, and decreased with increasing temperature. NH_4_^+^ ions were not detected above 620 °C. The amount of ammonia collected was determined by the following three factors. 1) The dissociation rate of nitrogen molecules by the Na-melt. 2) The reaction rate between dissociated nitrogen and hydrogen. 3) The decomposition rate of synthesized ammonia. In the ammonia decomposition process, a significant amount of ammonia is assumed to be decomposed before reaching the collecting solution because the flow rate of the supply gas is low (200 sccm), and, therefore, synthesized ammonia remains in the heated quartz tube for a long period.

Consequently, the reaction rates shown in Fig. 3 do not represent the equilibrium values of the ammonia synthesis reaction. According to previous reports, the nitrogen dissociation ability of Na is significantly increased above 600 °C, which suggests that the decrease in the reaction rate at increasing temperature was mainly due to the decomposition of synthesized ammonia before reaching the collecting solution^27^.

Several studies concerning the nitrogen dissociation ability of Na have been reported. In 1994, T. L. Bush *et al*. reported that a silicon nitride layer could be formed on Si substrates by heating a Si substrate coated with a monolayer of either Na or K metal to 500 °C in a nitrogen atmosphere^28, 29^. Furthermore, GaN crystals can be synthesized in a Ga-Na mixed melt at about 800 °C by pressurizing a nitrogen atmosphere to 30 atm. In this reaction, Na functions as a catalyst for nitrogen dissociation at the gas-liquid interface^27^. From these reports and our experimental results, we concluded that the Na-melt dissociates nitrogen molecules to facilitate their reaction with hydrogen in the present study.

Kitano *et al*. reported that the materials having low work function easily decompose the nitrogen molecules, which probably be one of the reason why Na functions as catalyst for ammonia synthesis because the work function of alkaline metals are remarkably small^9^.

The amount of Na^+^ ions detected decreased with increasing temperature up to 560 °C, and then increased rapidly with increasing temperature. The presence of Na^+^ ions in the collecting solutions was attributed to the dissolution of NaH formed by reaction with ammonia, because evaporated Na metal was collected in the cold-trap (0 °C) before reaching the collecting solutions.

A phase diagram of NaH constructed from first principle calculations was reported in 2006, and revealed that NaH is decomposed to Na metal and H_2_ above 425 °C at 1 atm^30^. The decrease in the amount of Na^+^ detected from 500 to 560 °C was attributed to the reduction in the synthesis rate of NaH resulting from the increased distance from the stable region of NaH. The rapid increase in the amount of Na^+^ detected above 590 °C was attributed to the reaction between Na vapor originating from the Na-melt and hydrogen in the low temperature region of the apparatus. This hydrogen may either be unreacted hydrogen from the supply gas, or have been generated by decomposition of synthesized ammonia.

As a result, in the case of synthesis temperature at 560 °C, ammonia could be synthesized without formation of NaH.

The conversion efficiency of hydrogen to ammonia was low as 0.0105% at the maximum, which seems to be the result of short dwell time of bubbles in liquid and small reactive surface area. However, confirmation of ammonia synthesis at ambient pressure will give us an opportunity to improve the reacting system. We are now designing an apparatus having a mechanics that can generates H_2_-N_2_ fine-bubble in large amount of molten-Na for improving the synthesis efficiency.

In this study, we demonstrated that molten Na metal works as a catalyst for the synthesis of ammonia from hydrogen-nitrogen mixed gas. Ammonia synthesis was achieved at ambient pressure and at a relatively low temperature of about 500 °C by supplying a 4%H_2_-96%N_2_ mixed gas into an Na-melt.

Using the molten metal catalyst will open a new possibility for ammonia synthesis.

## Methods

Na metal (20 g) was placed in a boron nitride (BN) vessel (inner diameter (I.D.): 22 mm, outer diameter (O.D.): 26 mm, height: 100 mm) in an Ar-purged glovebox. The BN vessel was transferred into a quartz tube (I.D.: 38 mm, O.D.: 40 mm), and connected to a flange with two nozzles for supplying and exhausting gas. The supply nozzle could be adjusted vertically. The quartz tube was set in a tubular furnace, and the supply nozzle was connected to a 4%H_2_-96%N_2_ gas cylinder. The exhaust gas was passed through a cold-trap into a collecting solution of methanesulfonic acid (1 mM).

After evacuating the quartz tube using a rotary pump, the 4%H_2_-96%N_2_ mixed gas was supplied until the inner pressure reached atmospheric pressure. The tubular furnace was heated to the desired temperature, then the supply nozzle was inserted into the molten Na metal while supplying the source gas. The source gas was supplied into the Na-melt at a flow rate of 200 sccm for 20 min, then the NH_4_^+^ ions trapped in the collecting solution were analyzed using ion chromatography. (ICS-5000, Thermo Fisher Scientific, Column type: 2 mmφ × 250 mm IonPac CS14, Detector type: thermal conductivity meter). In order to prevent raising the pressure in the system, suction pump was connected at the end of exhaust tube and vacuumed at the same rate of supplied gas.

In this synthesis process, the exhaust gas is heated to the same temperature as the Na-melt, because the electric furnace heats the entirety of the quartz tube. Therefore, some of the generated ammonia decomposes, until the exhaust gasses pass through the collecting solution.
